# Detection of multi-tomato leaf diseases (*late blight*, *target and bacterial spots*) in different stages by using a spectral-based sensor

**DOI:** 10.1038/s41598-018-21191-6

**Published:** 2018-02-12

**Authors:** Jinzhu Lu, Reza Ehsani, Yeyin Shi, Ana Isabel de Castro, Shuang Wang

**Affiliations:** 1 School of Mechanical Engineering, Xihua University, 999 Jinzhou Road, Chengdu, Sichuan 610000 China; 2 Mechanical Engineering Department, University of California-Merced, 5200 N. Lake Road, Merced, CA 95343 U.S.A.; 3 Department of Biological Systems Engineering, University of Nebraska-Lincoln, 3605 Fair Street, Lincoln, NE 68583 United States; 4 Department of Crop Protection, Institute for Sustainable Agriculture (IAS-CSIC), Cordoba, Spain

## Abstract

Several diseases have threatened tomato production in Florida, resulting in large losses, especially in fresh markets. In this study, a high-resolution portable spectral sensor was used to investigate the feasibility of detecting multi-diseased tomato leaves in different stages, including early or asymptomatic stages. One healthy leaf and three diseased tomato leaves (*late blight*, *target* and *bacterial spots*) were defined into four stages (healthy, asymptomatic, early stage and late stage) and collected from a field. Fifty-seven spectral vegetation indices (SVIs) were calculated in accordance with methods published in previous studies and established in this study. Principal component analysis was conducted to evaluate SVIs. Results revealed six principal components (PCs) whose eigenvalues were greater than 1. SVIs with weight coefficients ranking from 1 to 30 in each selected PC were applied to a *K*-nearest neighbour for classification. Amongst the examined leaves, the healthy ones had the highest accuracy (100%) and the lowest error rate (0) because of their uniform tissues. Late stage leaves could be distinguished more easily than the two other disease categories caused by similar symptoms on the multi-diseased leaves. Further work may incorporate the proposed technique into an image system that can be operated to monitor multi-diseased tomato plants in fields.

## Introduction

Fresh-market tomatoes are produced in every state in the US, where 20 states support commercial-scale production. According to data from the US Department of Agriculture, Florida is the leading state in fresh-market tomato production. However, a series of diseases has threatened tomato production in Florida, resulting in large losses in fresh and processed tomato production. Tomato diseases are caused by several factors, including fungal, bacterial and viral infections^1–4^. Most of the foliar diseases, such as *late blight*, *target* and *bacterial spots*, are favoured by warm temperatures or prolonged periods of wetness, which are typical in Florida. Conventional scouting for foliar diseases relies primarily on the visual inspection of leaf colour patterns and crown structures. Laboratory test approaches, such as polymerase chain reaction, enzyme-linked immunosorbent assay and loop-mediated isothermal amplification, are highly specific and sensitive to identify diseases on plant tissue samples. However, these approaches involve destructive methods, entail time- and labour-consuming processes and require specialised skills^5–8^.

Advancements in agricultural technology have offered opportunities for the non-destructive detection of plant diseases through spectroscopy^9^. A visible near-infrared spectrometer (400–1000 nm) is used in a reflectance mode to distinguish sprouted and intact wheat kernels under laboratory conditions^10^. Reflectance at 728 and 878 nm is utilised to classify sprouted and intact kernels, and the wavelength region above 720 nm is set to categorise sprouted kernels according to different levels of severity. The correct recognition rates of intact, sprouted and severely sprouted kernels are 100%, approximately 94% and 98%, respectively. The ability of reflectance spectroscopy in three regions, namely, ultraviolet, visible and near-infrared, has been evaluated indoors to determine the disease severity of tomato leaves infected with *Xanthomonas perforans*^11^. Partial least squares (PLS) regression, stepwise multiple logistic regression (SMLR) and their combinations have been applied to derive four predictive models. Amongst these four models, the model established by SMLR is the most efficient in predicting disease severity with a root mean square difference of 4.9% and a coefficient of determination of 0.82. Ground-level reflectance spectra have also been obtained for the in-field detection of plant nitrogen^12–14^. Spectral signatures of spectral data have been analysed in leaves to differentiate sugar beet diseases^15^. Spectral reflectance has been measured in fields by using a handheld spectroradiometer in the range of 400–1050 nm, and the highest correlation coefficients (r = 0.85) have been detected in the visible region. This study has also provided a basis for further development of classification methods of sugar beet diseases in different developmental stages.

Spectral vegetation indices (SVIs) from ground-level reflectance spectral data can be used to estimate crop yield^16^, detect variations in leaf area index^17^ and characterise agricultural crop biophysical variables^18^. SVIs can also be used to detect or differentiate various plant diseases^19–21^. Different diseases are often associated with specific physiological and visual changes in their host plants. Appropriate SVIs offer a great advantage in the dimension reduction of spectral data^22^. Spectral studies on detecting foliar diseases have been performed using destructive chemical methods^23–25^. Some studies have reported the use of non-destructive methods to detect foliar diseases on certain varieties. Few studies have focused on multi-diseased leaves with similar symptoms regardless of plant variety. Spectral data can be obtained using spectral sensors and hyperspectral imaging sensors. In agriculture, most spectral sensors are utilised to detect the internal qualities of fruits, such as sugar degree. Hyperspectral images are obtained to detect plant diseases, especially foliar diseases. Considering that diseases may cause symptoms on leaves, we may examine images to obtain a visual leaf image, especially when multiple scars are present on one leaf. Hyperspectral imaging technology requires large and time-consuming computation. Thus, this imaging is usually performed in laboratories for research involving detection approaches. In our previous work, we improved the possibility of using spectroscopy technology in plant disease detection. In the present study, we aimed to perform an in-depth research on the detection of multi-diseased leaves and promote spectroscopy technology on the detection of leaf diseases.

This research generally aimed to investigate the feasibility of a simple method to detect multi-diseased tomato leaves in different stages, including an early or asymptomatic stage, by using a spectral-based sensor. This study was specifically designed 1) to calculate and create SVIs from reflectance spectra that can describe healthy and diseased leaves in different stages; 2) to select SVIs consisting of multi-wavelengths that can mostly describe changes in leaves and 3) to establish and evaluate classification models on differentiating healthy, asymptomatic, early stage and late stage diseased plants by using the selected SVIs.

## Results

### Spectra of leaves

**Figure 1:**
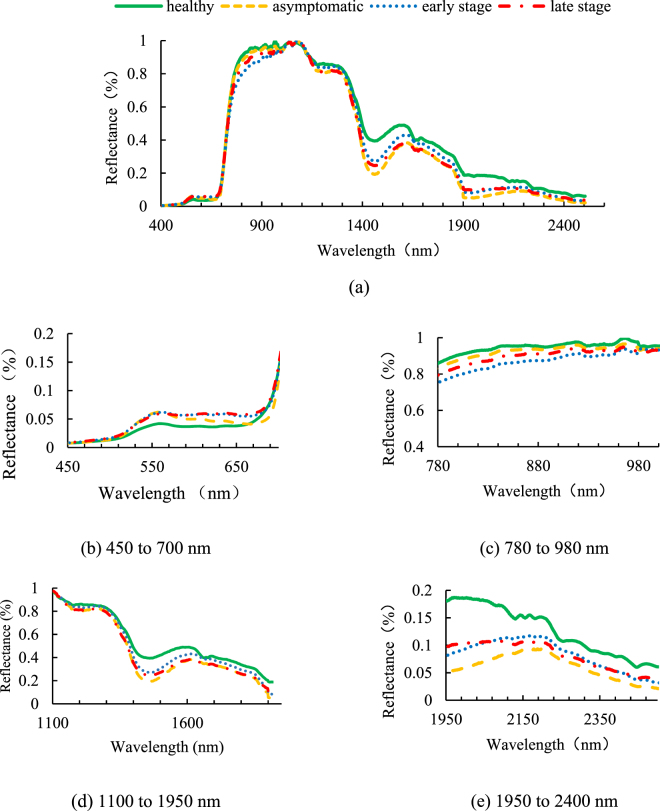
Average reflectance spectra of healthy, asymptomatic, early stage and late stage leaves: (**a**) spectral ranges of 400–2500 nm, (**b**) zoomed in spectral ranges of 450–700 nm, (**c**) zoomed in spectral ranges of 780–980 nm, (**d**) zoomed in spectral ranges of 1100–1950 nm and (**e**) zoomed in spectral ranges of 1950–2400 nm.

Figure 1 shows the averaged reflectance spectra of all of the data after a baseline correction was performed [Equation (1)]. These four spectra significantly differ in several bands in the whole wavelength (Fig. 1). To observe the spectra in detail, we zoomed in and divided four reflectance spectral bands into one visible band and three near-infrared bands [Fig. 1(b) to (e)].

Figure 1(b) illustrates the zoomed reflectance spectra at 450–700 nm. Compared with the spectrum of the healthy leaf, the spectra of three other leaf diseases increased in reflectance from 500 nm. In the range of 500–570 nm, these three curves were intersected. Beyond 570 nm, two symptomatic diseased leaves (‘early stage’ and ‘late stage’) remained intersected, and both were higher than the two other categories. The average reflectance of the asymptomatic leaves decreased from 570 nm and then kept lower than that of the two symptomatic leaves. The reflectance of the asymptomatic leaves was retained at levels higher than that of the healthy ones from 660 nm and beyond. All of the spectra increased suddenly at around 690 nm. Figure 1(c) shows the zoomed reflectance spectra at 780–1000 nm. The reflectance spectra of the four categories represented regular changes. The reflectance spectra of healthy, asymptomatic, early stage and late stage were in a decreasing trend. All of the spectra exhibited a flattened slope, whose reflectance was not smaller than 75% and not greater than 100%. Figure 1(d) illustrates the zoomed in reflectance spectra at 1100–1950 nm. All of the reflectance spectra declined from 1100 nm to around 1400 nm in one bottom section in the region. The change in the four spectra was gradually irregular. Figure 1(e) shows the zoomed in reflectance spectra in 1950–2400 nm. The reflectance spectra of the healthy leaves were significantly higher than those of the three diseased leaves. The spectral curves of the asymptomatic and early stage leaves showed a similar trend, which peaked at around 2200 nm, and declined slowly until 2500 nm was reached. The spectra of the late stage leaves had two intersection points with those of the early stage leaves at 2050 and 2390 nm.

### Spectral vegetation indices by PCA

Fifty SVIs from previous studies and seven SVIs obtained in this study were subjected to principal component analysis (PCA). Their eigenvalues were used as assessment criteria. An eigenvalue of less than 1 for one principal component (PC) of an SVI indicates that the effect of such a PC is less important than single variable. Therefore, only PCs with eigenvalues greater than 1 were chosen for the next step in this study. In each chosen PC, the SVIs were arranged according to their weight coefficient starting from the highest to the lowest. A total of 147 samples were included in one category in this test. Considering the principle ‘sample number is 5–10 times greater than the optimal feature number,’ we used the minimum and maximum numbers of features as two cut points, whose components were scored in a column with a list of the first 15 and 30. Table 1 shows the PCs and the spectral indices combinations. Furthermore, 6 eigenvalue roots of the PCs were greater than 1. In each of the 6 PCs, 15 and 30 SVIs were found. The SVIs with the highest frequency were PSSR_c_, NDWI-Hyp, mSRI_3_ and BRI_2_. The SVIs with a frequency of less than 1 were SIPI, ND_5_, NDVI, ND_1_, SR_1_ and RDVI. Considering the low frequency, we found that the SVIs were slightly correlated with classification, and only SVIs ranking from 1 to 30 were selected as input in the classifier for further data analysis.

**Table 1:** PCs with different eigenvalues and their component SVIs ranking from 1 to 30.

PCs	Eigenvalue	SVIs rank 1–15	SVIs rank 16–30
PC1	25.521	ND_1_, CTR_2_, ND_5_, SR_3_, SIPI, SR_6_, NDVI, MSR_2_, SR_5_, SR_4_, ND_2_, ND_3_, ND_4_, mND, mSRI_3_	LIC, BRI_1_, DSWI_5_, VOG, SR_7_, PSSR_c_, NDWI, WI, RGRI, BRI_2_, PSSR_b_, BI, CTR_1_
PC2	13.880	BGI_1_, SR_1_, BGI_2_, SR_2_, CI_1_, DSWI_2_, RDVI, MTVI, AVE, TVI, DSWI_4_, DSWI_3_, MCAI, CI_2_, GREEN I	PRI_2_, PSSR_b_, REP, PRI_1_, PRI_3_, TCARI, RGRI, MCARI, BI, NDWI-Hyp, PSSR_c_, VOG, SR_7_, BRI_2_, ND_2_
PC3	6.398	ARI, MCARI, TCARI, MSI, TVI, DSWI2, MCAI, AVE, MTVI, DSWI_1_, REP, RDVI, NDWI, WI, NDWI-Hyp	DSWI_5_, SR_1_, PSSR_c_, SR_2_, BRI_2_, PSSR_b_, CTR_1_, RI, RGRI, mSRI_1_, mSRI_3_, BGI_1_, BRI_1_, SR_5_, mSRI_2_
PC4	5.194	RI, CI_2_, CTR_1_, PRI_1_, SR_7_, VOG, RE, CI_1_, BRI_2_, GREEN I, NDWI-Hyp, PSSR_c_, BGI_2_, MSRI_3_, MSR_1_,	mND, SR_4_, ND_3_, ND_4_, REP, ARI, MCARI, DSWI_3_, DSWI_4_, SR_5_, TCARI, RDVI, LIC, MTVI, mSRI_2_
PC5	2.057	NDWI-Hyp, DSWI_1_, CTR_1_, BRI_1_, WI, BRI_2_, MSI, NDWI, DSWI_5_, PRI_3_, PRI_2_, LIC, RGRI, MSRI_3_, MSR_1_,	DSWI_3_, DSWI_4_, ARI, SR_1_, SR_2_, GREEN I, MCAI, PSSR_c_, TVI, SR_3_, CTR_2_, MTVI, AVE, BI, CI_1_
PC6	1.080	BI, PRI_2_, LIC, NDWI, PRI_3_, BGI_1_, MCAI, RI, DSWI_1_, RE, VOG, SR_7_, BGI_2_, WI, SR_4_	ND_4_, ND_3_, BRI_1_, CI_1_, mND, DSWI_5_, MSR_1_, MSRI_3_, TVI, MTVI, AVE, MSI, CTR_2_, NDWI-Hyp, RDVI

### Classification

For each sample, two SVI combinations were used for classification. One combination involved SVIs ranking from 1 to 15, and the other combination comprised SVIs ranking from 1 to 30. The classification results of the two datasets were compared to evaluate the effects of the number of SVIs. Both datasets were divided into training and testing groups based on the ratio of 70%:30%. For instance, the error rate of ‘C’ was C samples misclassified into other categories divided by all C samples. The accuracy of ‘C’ is correctly classified as C samples in all of the C samples. The training set was treated as a known group because KNN used lazy learning, and the classification results consisted of accuracy and error rate based on the testing group. Table 2 summarises the error rate of the healthy, asymptomatic, early stage and late stage leaves. Based on the error rate of the testing group, the highest error rate of the four categories with different PCs resulted in 30 SVIs. In the six PCs, the lowest error rates of healthy, asymptomatic, early stage and late stage leaves did not have significant differences, which were 0, 4.8, 5.6 and 0, respectively. However, classification difficulties observed when 30 SVIs of healthy, asymptomatic, early stage and late stage leaves were used, and the error rates were 26.9% in PC1, 28.6% in PC1 or PC3, 23.3% in PC4 and 25% in PC1. The asymptomatic leaves could be classified as the worst amongst the three other leaves, while the three other categories were categorised correctly at least in one PC combination.

**Table 2:** Error rates of classifying healthy, asymptomatic, early stage and late stage leaves with six PCs and two SVI compositions.

PCs	Healthy		Asymtomatic		Early stage		Late stage	
	15SVIs	30SVIs	15SVIs	30SVIs	15SVIs	30SVIs	15SVIs	30SVIs
PC1	11.8	26.9	9.5	28.6	19.5	7.3	1	25
PC2	0	0	19	16.1	7.7	12.5	7.9	4.7
PC3	8.3	0	9.5	28.6	5.6	13.9	9.5	0
PC4	25	0	17.8	13	0	23.3	15	0
PC5	12.5	0	21.7	4.8	6.8	10.2	2.9	2.3
PC6	0	0	9.5	25	11.1	7.8	5.6	8.3

Table 3 summarises the accuracy of healthy, asymptomatic, early stage and late stage leaves. Each of the six PCs contained two SVI combinations. A total of 12 results were included in one category. Based on the accuracies of the testing group, the highest accuracies of the healthy, asymptomatic and late stage leaves were 100%. By comparison, the highest accuracies of different PC or SVIs of early stage leaves ranged from 73.5% to 97.8%. In different PCs or two SVI combination groups, the lowest accuracies of the healthy, asymptomatic, early stage and late stage leaves were 85.7%, 65.2%, 73.5% and 77.1%, respectively. The difficulties in the classification of the asymptomatic leaves occurred in PC1 when 30 SVIs were used, and the accuracy was 65.2%. In the three other categories, the accuracies were higher than 70%.

**Table 3:** Classification results of healthy, asymptomatic, early stage and late stage leaves with six PCs and two SVIs compositions.

PCs	Healthy		Asymtomatic		Early stage		Late stage	
	15SVIs	30SVIs	15SVIs	30SVIs	15SVIs	30SVIs	15SVIs	30SVIs
PC1	100	90.5	95	65.2	84.6	75	77.1	90.9
PC2	95.7	100	89.5	92.9	92.3	85.4	89.7	91.1
PC3	100	100	86.4	87.0	89.5	81.6	92.7	91.4
PC4	85.7	100	100	76.9	73.5	94.3	100	85.3
PC5	100	100	90.0	83.3	85.4	97.8	91.7	95.5
PC6	100	100	86.4	85.7	93.0	90.4	91.9	86.8

## Discussion

### Spectral analysis

In the visible range, the diseased leaves, especially those with symptoms, had spots or scars. The symptoms of these three diseases are light or dark brown spots on the leaf. The colour induced less absorption in the diseased leaves in the reflectance mostly in the green range (495–570 nm) and in the red edge range (around 690 nm). As an indicator of plant chlorophyll content^26,27^, the red edge (680–750 nm) differed in the spectrum slope. The effect of disease on leaf wetness, which indicates the relationship of infection with water content, has been studied^28,29^. Laboratory studies on leaf water content have been conducted in the near infrared region of the leaf spectra (780–2500 nm)^30–32^. The average spectra of the asymptomatic and healthy leaves were compared, and the results demonstrated that the two other symptomatic leaves exhibited a strong spectral change in the entire spectral region.

In the spectral range of 1000 nm and above, the reflectance of the four categories of leaves did not significantly change, and this condition may be observed in multiple diseases. Some studies have reported that plant cellular contents, such as enzymes and proteins, which represent the changes in the reflectance spectra in the range of 1000–2500 nm, are affected by specific pathogens^33^. Each pathogen causes specific changes in the spectra that show regular pathogenic patterns. In our experiments, the diseased leaves were collected from a field and inoculated with pathogens in the natural environment. A certain pathogen in each leaf was difficult to control, and the disease consequently spread in one area. Enzymes elicit various effects on different diseases. Moreover, we classified the leaves according to the disease stage instead of the varieties of the diseases. As such, changes in the multi-diseased leaves were irregular in the spectral range exceeding 1000 nm.

### SVI calculation

The majority of SVIs were sourced from previous studies, whereas seven SVIs were established in this study based on the analysis of the spectral reflectance. After PCA was conducted, each of the 30 SVIs of the 6 PC combinations was selected. Four SVIs with the highest frequency (PSSRc, NDWI-Hyp, mSRI_3_, BRI_2_) and six SVIs (SIPI, ND_5_, NDVI, ND_1_, SR_1_ and RDVI) were not observed in different PC combinations. These high-frequency SVIs were associated with the wavelengths in the green region (445 and 450 nm), red and far-red region (690, 707 and 750 nm) and near-infrared region (800, 1070 and 1200 nm). However, the SVIs that were not selected for PCA had the same wavelengths at 800 nm (SIPI, NDVI, ND_1_, SR_1_ and RDVI) and 750 nm (ND_5_). In these two wavelengths, significant differences in the spectra were found between the healthy and diseased leaves and between the leaves in two disease stages. However, the spectra in this study were all averaged in at least 74 spectra. Several spectra were detected above or below the average. Therefore, one disease or even multi-diseased leaves could be inadequately described on single wavelengths. This study also explained why two SVIs (ND_5_ and mSRI_3_) created by some wavelengths in this study differed from the selected SVIs. High-frequency SVIs, such as PSSR_c_, mSRI_3_ and BRI_2_, were related to pigments, such as carotenoids and chlorophyll. Visual diseased spots on the leaves were caused by pigment losses. However, asymptomatic leaves varied from the expected finding except the disrupted plant cells^34^, and several diseases affect leaves even in the asymptomatic stage. Low-frequency SVIs, such as NDVI, SIPI and RDVI, and spectral ratios are highly related to plant vigour or pigments in remote sensing studies^35,36^, especially on nitrogen detection or soil status by SVIs^37^. In terms of foliar disease stages, these SVIs were poorly correlated with classification in this study.

### Classification

When the plants were affected by diseases in this study, especially in the late stage, visual spots or scars were found on the leaves. Amongst the diseased leaves, the late stage leaves showed the highest classification accuracy and were the least affected by the number of two SVIs, whose accuracy was 14.7%. In experiment sampling, regions of interests (ROIs) were selected mainly around the lesions. The heavier the tissue was, the greater the differences between healthy tissues and diseased spots would be. Therefore, the late stage leaves could be distinguished more easily than the two other disease categories. The accuracies of the early stage and asymptomatic leaves were lower than those of the leaves in other stages. The accuracy of the asymptomatic leaves was higher than that of the early stage leaves, whereas the error rate of the asymptomatic leaves was lower than that of the other leaves. This misjudgement might be attributed to the confusion between asymptomatic and healthy leaves, that is, when the plants were initially affected by the disease, the viruses or bacteria existed in tissues or cells. Pathogens usually undergo a long incubation period before symptoms can be observed. Amongst the four categories in this study, the healthy leaves had the highest accuracy (100%) and the lowest error rate (0) mainly because of sampling. The spectroscopy sensor had a 4° field of view (FOV), and the distance between the sensor and the leaves was about 50 cm. Therefore, the ROIs could not be concentrated at a certain point. When the healthy leaves were sampled, the tissues were all healthy regardless of ROIs. By comparison, the regions of the tissues of the healthy and diseased areas on the diseased leaves overlapped. Consequently, the spectral data of the diseased leaves did not completely represent the diseased categories. Nevertheless, the highest accuracy of classifying the healthy, asymptomatic and late stage leaves was 100% when different PCs and SVIs were used, thereby confirming the feasibility of using the spectroscopy-based sensor with SVIs for the classification of tomato multi-disease leaves with similar symptoms even in the asymptomatic stage. Further studies should incorporate the proposed technique into an image system that can be operated to monitor multi-diseased tomato plants in fields.

## Methods

### Spectral Sensor

A portable high-resolution SVC HR-1024 spectroradiometer (Spectra Vista Corporation, Poughkeepsie, NY, USA) was used to collect reflectance data in the range of 350–2500 nm, with spectral resolutions of less than or equal to 3.5, 9.5 and 6.5 nm at the wavelength ranges of 350–1000, 1000–1850 and 1850–2500 nm, respectively. The spectral data were collected using a 4° FOV lens at a minimum integration time of 4 ms. To acquire the relative reflectance spectra of the sample, we conducted a correction by using Equation (1). The raw spectra (R_sample_) were calibrated in terms of white (R_reference_) and dark correction (R_dark_). The white correction was obtained with a white reference panel (Spectralon Reflectance Target, CSTM-SRT-99–100 Spectra Vista Corporation, Poughkeepsie, NY, USA), and the dark correction was carried out by covering the lens with a light-proof cap.

1 $$R=\frac{{R}_{sample}-{R}_{dark}}{{R}_{reference}-{R}_{dark}}$$

### Diseases

**Figure 2:**
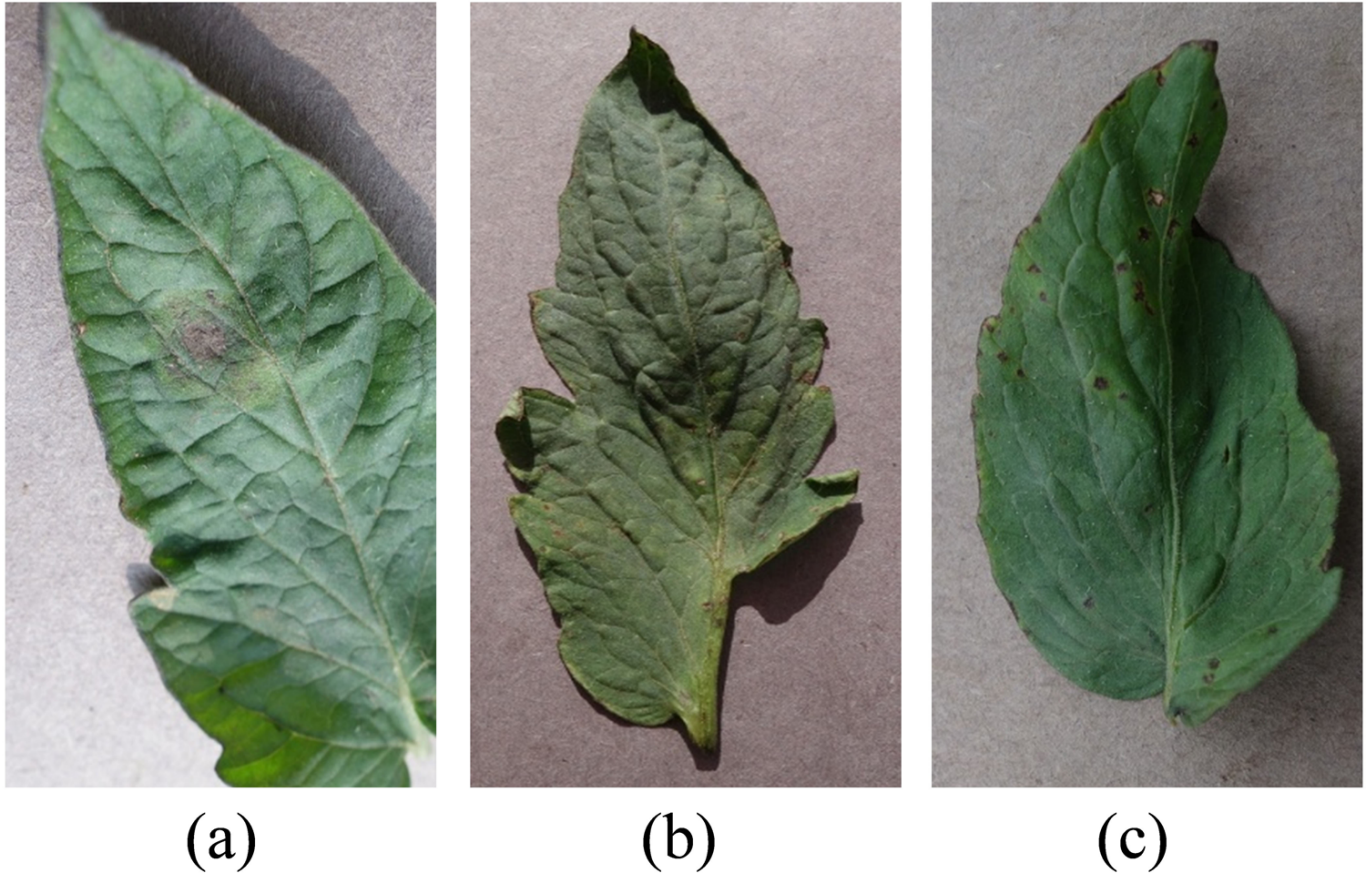
Three diseased tomato leaves with disease symptoms: (**a**) *Late blight*; (**b**) *Target*; (**c**) *Bacterial spot*. Original source: Plant Village^43^.

*Late blight*, *target* and *bacterial spot* are three widely diseases that spread in tomatoes produced in Florida. In Fig. 2, all of these diseases manifested similar symptoms: irregular yellow necrotic areas, irregularly shaped water-soaked or brown spots and lesions on tomato leaves. The infected plants manifest necrotic crowns and die^38^. Infection caused by one pathogen may cause plants to become susceptible to infection or colonization by other pathogens, leading to multi-diseased leaves. With these adverse conditions, tomato diseases should be detected in early stages in fresh markets.

### Sampling

Experiment was conducted in a commercial farm in Naples, Florida. All of the leaves were collected by professional experts, who defined the diseased stages: ‘asymptomatic’ (leaves from the diseased plant without foliar symptoms that can be observed by human eyes), ‘early stage’ (symptomatic leaves from diseased plants that can be cured by fungicides) and ‘late stage’ (symptomatic leaves from incurable diseased plants). With warm temperatures or periods of wetness in Florida, diseases spread fast and induce cross infections. Multi-diseased leaves are commonly seen in actual fields. Other studies on tomato diseases usually involve artificial inoculation method or strict detachment of leaves to ensure that only one disease affects one leaf. In our experiment, multi-diseased leaves were selected in an uncontrolled cultivation environment, which usually spread three diseases, namely, *late blight*, *bacterial spot* and *target*. Multi-diseased leaves from plants in each disease stage have one or two characteristic symptoms of a particular disease. Therefore, we classified the leaves based on leaf stage instead of disease varieties. *Late blight* was observed in the asymptomatic stage, *bacterial spot* and *late blight* were detected in early stage, and *bacterial spot* and *target* were found in late stage.

**Figure 3:**
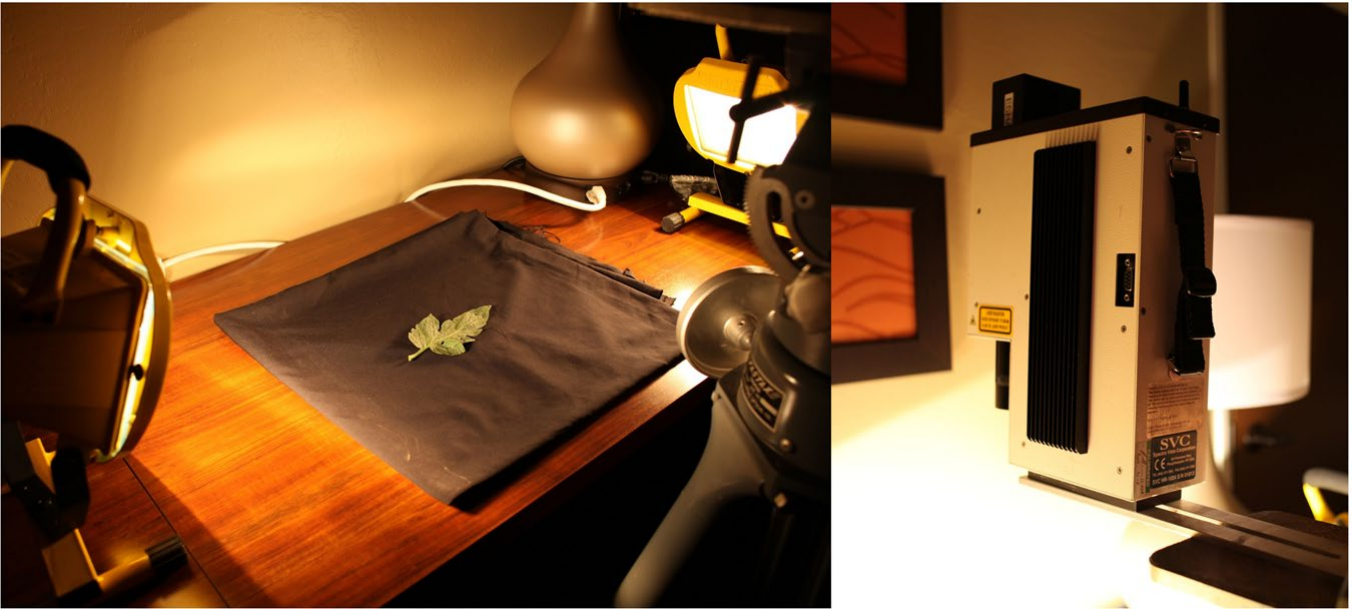
Detection system in our laboratory.

In each stage, three or five leaves were collected from one plant in each disease stage. Each leaf sample was placed in an isolated plastic bag immediately stored in an icebox. Measurements were performed within 3 h after the leaves were collected. The proposed detection system was composed of the sensor and two 500 W halogen portable lights from two symmetric sides to provide a uniform light condition (Fig. 3). At the start of each measurement, other lights were turned off to ensure that no any interference light was present during the experiment. The leaves were placed in a fixed plate in a black background. The sensor was installed 45–50 cm high from the plate. Three measurements were conducted in one leaf containing three different spots on one leaf to obtain an average spectrum in the dataset. After sampling was completed, the spectra were checked manually. Any spectrum with interfering signals was removed before it was used as an input of the dataset. The spectral data of the corresponding sample were collected again. The dataset consisted of 74, 77, 148 and 146 leaf spectra for healthy, asymptomatic, and symptomatic categories, respectively.

### Spectral vegetation indices

SVI is used to enhance the characteristics of information, such as the object’s properties, based on two or more wavelengths or range of wavelength reflectance combination. Seven kinds of vegetation indices, namely, broadband of green, narrow strip of green, light canopy of utilization, nitrogen, drought or carbon decay, pigment and canopy water content, were achieved. We listed 50 normally used SVIs and 7 SVIs created in this study (Table 4). These SVIs in the wavelengths of the visible and near-infrared regions were calculated from the raw spectra after baseline correction and outlier removal were performed.

**Table 4:** List of spectral vegetation indices cited in previous studies and established in this study.

SVIs	Formula	Literature
Simple ratio	SR_1_ = R_800_/R_550_	^44^
	SR_2_ = R_750_/R_550_	^44^
	SR_3_ = R_800_/R_680_	In this study
	SR_4_ = R_750_/R_700_	In this study
	SR_5_ = R_800_/R_670_	^45^
	SR_6_ = R_795_/R_670_	In this study
	SR_7_ = R_740_/R_720_	In this study
Green indices	Green I = R_570_/R_670_	^36^
Disease–Water Stress Index	DSWI_1_ = R_800_/R_1660_	^46^
	DSWI_2_ = R_1660_/R_550_	^46^
	DSWI_3_ = R_1660_/R_680_	^46^
	DSWI_4_ = R_550_/R_680_	^46^
	DSWI_5_ = (R_800_ + R_550_)/(R_1660_ + R_680_)	^46^
Anth reflectance index	ARI = 1/R_550_ − 1/R_700_	^47^
Moisture stress index	MSI = R_1600_/R_820_	^48^
Blue indices	BI = R_450_/R_490_	^49^
Pigment specific simple ratio chlorophyll b	PSSR_b_ = R_800_/R_635_	^50^
Pigment specific simple ratio carotenoids	PSSR_c_ = R_800_/R_500_	^50^
Carotenoid indices	CI_1_ = R_515_/R_570_	^51^
	CI_2_ = R_520_/R_500_	^51^
Water index	WI = R_900_/R_970_	^52^
Red edge	RE = R_750_/R_710_	^22^
Vogelmann	Vog = R_740_/R_720_	^53^
Redness index	RI = R_700_/R_670_	^54^
Normalized difference vegetation index	NDVI = (R_800_ − R_670_)/(R_800_ + R_670_)	^55^
Normalized difference	ND_1_ = (R_800_ − R_680_)/(R_800_ + R_680_);	^56^
	ND_2_ = (R_750_ − R_660_)/(R_750_ + R_660_)	^44^
	ND_3_ = (R_750_ − R_705_)/(R_750_ + R_705_)	^44^
	ND_4_ = (R_755_ − R_705_)/(R_755_ + R_705_)	In this study
	ND_5_ = (R_680_ − R_500_)/ R_750_	In this study
Modified ND	mND = (R_750_ − R_445_)/(R_705_ + + R_705_ − R_445_)	^57^
Modified simple ratio	mSR_1_ = (R_750_ − R_445_)/(R_705_ + R_445_)	^56^
	${{\rm{mSRI}}}_{2}=\frac{({R}_{800}-{R}_{670})-1}{\sqrt{({R}_{800}+{R}_{670})}+1}$	^56^
	mSRI_3_ = (R_750_ − R_445_)/(R_750_ + R_445_)	In this study
NDWI-hyperion	NDWI-Hyp = (R_1070_ − R_1200_)/(R_1070_ + R_1200_)	^58^
ND water index	NDWI = (R_860_ − R_1240_)/(R_860_ + R_1240_)	^59^
Structure-intensive pigment index	SIPI = (R_800_ − R_445_)/(R_800_ + R_680_)	^60^
Photochemical reflectance index	PRI_1_ = (R_515_ − R_531_)/(R_515_ + R_531_)	^61^
	PRI_2_ = (R_534_ − R_565_)/(R_534_ + R_565_)	^62^
	PRI_3_ = (R_530_ − R_570_)/(R_530_ + R_570_)	^63^
Modified chlorophyll absorption in reflectance index	MCARI = [(R_700_ − R_670_)–0.2*(R_700_ − R_550_)]/(R_700_/R_670_)	^64^
Transformed chlorophyll absorption in reflectance index	TCARI = 3*[(R_700_ − R_670_)–0.2*(R_700_ − _550_)/(R_700_/R_670_)]	^65^
Renormalized difference vegetation index	${\rm{RDVI}}=\frac{({R}_{800}-{R}_{670})}{\sqrt{({R}_{800}+{R}_{670})}}$	^66^
Triangular veg. index	TVI = 0.5* 120*(R_750_ − R_550_) − 200* R_670_ − R_550_)	^67^
Average in the range of 750~850 nm	AVE = average between R_750_ and R_850_	^64^
Modified chlorophyll-absorption-integral	${\rm{mCAI}}=\frac{({R}_{752}+{R}_{545})({R}_{752}-{R}_{545})}{2}-{\sum }_{545\,nm}^{752\,nm}(R\ast 1.158)$	^68^
Red green ratio index	${\rm{RGRI}}=\frac{{\sum }_{i=635\,nm}^{695\,nm}R}{{\sum }_{i=495\,nm}^{555\,nm}R}$	^69^
Red edge position	REP = 700 + 40 (R_RE_ − R_700_) /(R_740_ − R_700_)	^70^
Blue green pigment indices	BGI_1_ = R_400_/R_550_	^71^
	BGI_2_ = R_450_/R_550_	^71^
Blue red pigment indices	BRI_1_ = R_400_/R_690_	^71^
	BRI_2_ = R_450_/R_690_	^71^
Lichtenthaler indices	LIC = R_440_/R_740_	^72^
Carter Indices	CTR_1_ = R_695_/R_420_	^73^
	CTR_2_ = R_695_/R_760_	^73^
Modified triangular vegetation index	MTVI = 1.2*(1.2*(R_800_ − R_550_) − 2.5*(R_670_ − R_550_))	^74^

### Principal Component Analysis (PCA)

PCA is a commonly used multivariate statistical method that simplifies datasets. In orthogonal transformation, the observations of possibly correlated variables were converted into a set of values of linearly uncorrelated variables called principal components (PC). In geometry, this transformation is equivalent to coordinating selection or translation in a multidimensional vector space. In algebra, this transformation corresponds to solving a feature value of a covariance matrix to obtain new variables (axis). According to the size order of variance (feature value), these variables become the first main components (PC1), the second main components (PC2), the third main components (PC3), and so on, indicating that PC1 has the largest possible variance. The main idea of PCA is data dimension reduction. The number of new variables is less than or equal to the number of original variables. In PCA, a weight coefficient of one new variable represents the importance of the new variable.

**Figure 4:**
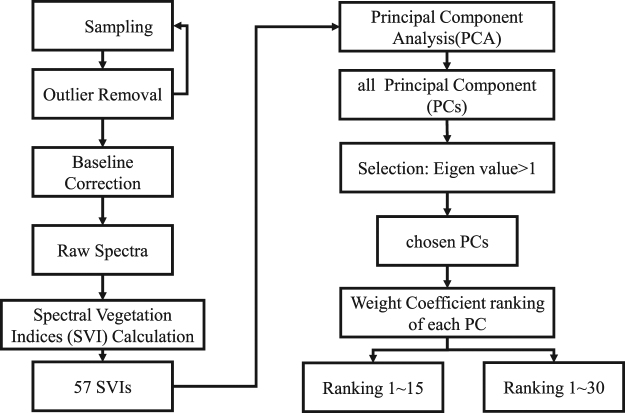
Flowchart of data processing.

PCA is commonly used to select sensitive wavelengths in spectral analysis. However, according to its principle, one component (PC) consists of more than one wavelength. Using one wavelength may lack persuasiveness for further classification. In this study, combining SVIs and PCA could solve the problem. The flowchart of data processing is shown in Fig. 4. We subjected the calculated 57 SVIs to PCA and used the eigenvalue as the assessment criteria. Only PCs with eigenvalues greater than 1 were then chosen for the next step. In each selected PC, the SVIs were arranged according to their weight coefficient starting from the highest to the lowest. Each PC consisted of 57 variables (SVIs) with an individual weight coefficient that can represent the importance of one variable. However, using one variable is similar to utilising one wavelength. As such, it may lack persuasiveness for further classification. Therefore, numerous SVIs, which consisted of multi-wavelengths, were selected in one PC in this work. Furthermore, for each sample, two SVI combination groups were applied to compare the classification. According to the principle, ‘sample number is 5–10 times greater than the optimal feature number’, the minimum and maximum numbers of features were treated as two cut points, whose components were scored in a column that was listed for each PC. Thus, the effects of the number of SVIs can be evaluated in terms of their classification results.

### K-Nearest Neighbour (KNN)

Classification analyses were conducted in PASW Statistics 18 (The SPSS Inc., Quarry Bay, Hong Kong). The *K*-nearest neighbour (KNN) used in this study is a simple classifier that works well on basic recognition problems in machine learning techniques^39,40^. Classification is achieved by identifying the nearest neighbours to a query example and then using such neighbours to determine the class of the query. This method is easily implemented and can obtain good results if the neighbours (k value) are chosen carefully.

In this work, we obtained a small dataset of 74, 77, 148 and 146 healthy, asymptomatic, early and late stage categories of diseased tomato leaves, respectively. According to our previous work^41,42^, KNN is an optimal classifier for the detection of diseases in indoor plants with a sample size of less than 200. Considering the continuity and the objectives of our work, KNN was used for further classification.

In the first step, the sample distance between the unspecified group and the known group is calculated. Then, the k neighbours of the unspecified samples at a certain distance are located. If these k neighbours belong to one group, the sample is assigned to the category. If more than one group is found in the k neighbours, the sample is included in the major category. In this paper, the sample distance was calculated by Mahalanobis distance method because the samples were numerical [Equation (2)]:

2 $${\rm{d}}({X}_{i},\,{X}_{j})=||{X}_{i}-{X}_{j}||={({\sum _{k}^{m}{W}_{k}|{X}_{ik}-{X}_{jk}|}^{2})}^{\frac{1}{2}}$$

where *X*_*i*_ and *X*_*j*_ are two samples in the dataset; *n* is the number of sample properties; *W* is the category in which *X*_*i*_ or *X*_*j*_ is assigned.

When k has a different value, the results change. A small k value may cause overfitting amongst the data in the model, whereas a large k value requires a long computation time. The k values selected in this study were 3, 4 and 5. The best k value was determined through cross-validation.

### Classification

The accuracy and error rate are determined by equations (3) and (4).

3 $${\rm{accuracy}}=\frac{correctly\,classified\,leaves\,samples\,in\,C}{all\,leaves\,samples\,in\,C}$$

4 $${\rm{error}}\,{\rm{rate}}=\frac{leaves\,C\,samples\,misclassified\,into\,other\,cathgories}{all\,leaves\,samples\,in\,C}$$

where C represents the categories: ‘healthy’, ‘asymptomatic’, ‘early stage’ and ‘late stage’.

The classification steps are as follows:

Step 1: Randomly divide the SVIs of all of the leaf samples into a training set and a testing set according to the ratio of 70%:30%.

Step 2: Determine the category of each sample in the training set by lazy learning, such KNN, so they are treated as known groups.

Step 3: Randomly choose a new leaf sample in the testing set.

Step 4: Calculate the Mahalanobis distance from the new leaf sample to each sample in the known groups.

Step 5: According to the distance, select KNN around the new leaf sample.

Step 6: Choose the highest frequency of 4 categories amongst KNN and determine the new sample category.

Step 7: Repeat steps 3 to 6 until no more samples exist in the testing set.

Step 8: Calculate the accuracy and the error rate.

## Electronic supplementary material

plant cultivation farm
