# Supplementary material for: Detection of multi-tomato leaf diseases (late blight, target and bacterial spots) in different stages by using a spectral-based sensor
Source: Sci Rep. 2018 Feb 12;8:2793. doi: 10.1038/s41598-018-21191-6 (PMC5809472; doi:10.1038/s41598-018-21191-6)
Supplement: Supplementary file 1 — plant cultivation farm [file 41598_2018_21191_MOESM1_ESM.docx]

Detection of multi-tomato leaf diseases (*late blight, target and bacterial spots*) in different stages by using a spectral-based sensor

Jinzhu Lu ^1^*, Reza Ehsani^2^, Yeyin Shi ^3^ , Ana Isabel de Castro ^4^ and Shuang Wang ^1^

^1^ School of Mechanical Engineering, Xihua University, 999 Jinzhou Road, Chengdu, Sichuan, 610000, China;

^2^ Citrus Research and Education Center, University of Florida/IFAS, 700 Experiment Station Road, Lake Aflred, FL 33850, United Stataes of America;

^3^ Department of Biological Systems Engineering, University of Nebraska-Lincoln, 3605 Fair Street, Lincoln, NE 68583, United States;

^4^ Department of Crop Protection, Institute for Sustainable Agriculture (IAS-CSIC), Cordoba, Spain;

* Correspondence: lujingzhu1103@163.com; Tel.: +86-28-87729082


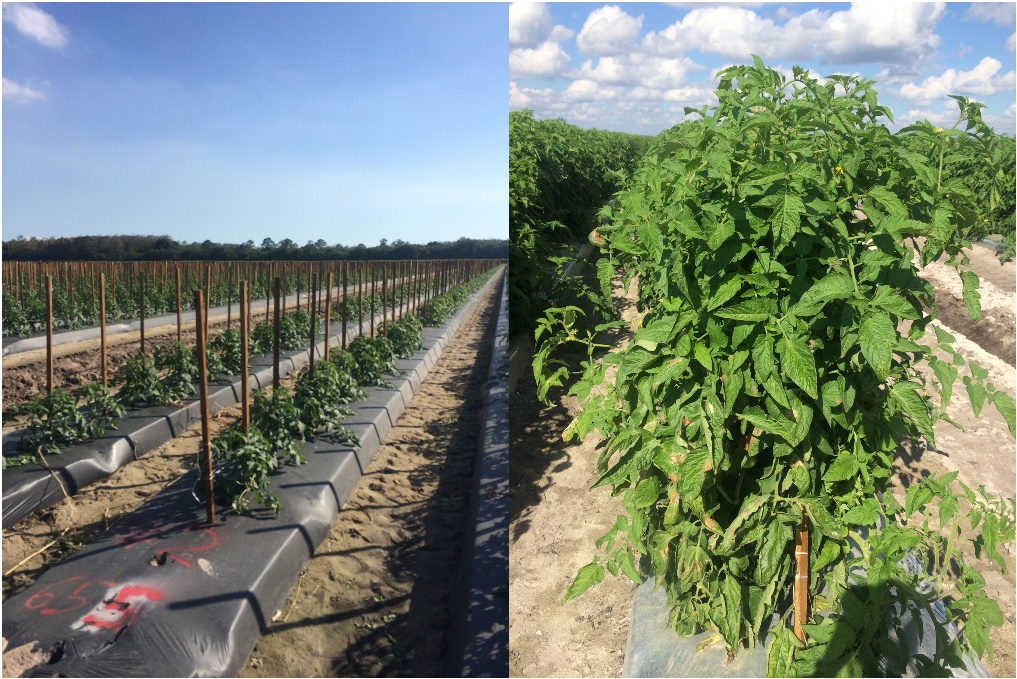


Plant cultivation farm
